# Antibacterial, antioxidant and cell proliferative properties of *Coccinia grandis fruits*

**Published:** 2017

**Authors:** Prashant Sakharkar, Balwantsinh Chauhan

**Affiliations:** 1 *Department of Clinical & Administrative Sciences, Roosevelt University College of Pharmacy, 1400 N. Roosevelt Blvd., Schaumburg, IL, 60173, USA*; 2 *Department of Bipoharmaceutical Sciences, Roosevelt University College of Pharmacy, 1400 N. Roosevelt Blvd., Schaumburg, IL, 60173, USA*

**Keywords:** Coccinia grandis, Antibacterial, Antioxidant, Cell proliferation, Ivy gourd

## Abstract

**Objective:**

Little knowledge is available on the antimicrobial and antioxidant properties of *Coccina grandis* fruits and no study has reported on its cell proliferative property. The aim of this study was to examine the antimicrobial, antioxidant and cell proliferative property of fruits of *C. grandis*.

**Materials and Methods:**

Fruits of *C*. *grandis* were extracted using water; ethanol and acetone by cold and hot Soxhlet extraction. The antibacterial activities of the extracts were tested against *Staphylococcus aureus, Enterococcus faecalis, Escherichia coli *and* Pseudomonas aeruginosa* using the modified Kirby-Bauer diffusion method and compared against erythromycin. The antioxidant property was determined using Cayman's antioxidant assay; whereas cell proliferation/cytotoxic properties were evaluated using the Cell Titer 96 Aqueous One Solution Cell MTS assay with MDA-MB 321 breast cancer cells. Data were analyzed for correlation and differences using unpaired student's t-test and one-way ANOVA. A p value of <0.05 was considered statistically significant.

**Results:**

Both cold and hot ethanol and acetone extracts of *C. grandis* fruits showed some degree of bacterial growth inhibition. Acetone extracts exhibited higher antibacterial activity. Both ethanol extracts showed antioxidant property when compared with standard Trolox. In contrary to cytotoxicity, all four extracts showed cell proliferation compared to controls at different concentrations. However, acetone extracts exhibited greater cell proliferation compared to ethanol extracts and cold extracts performed better than the hot extracts.

**Conclusion:**

*C. grandis* fruits exhibited some degree of antimicrobial, antioxidant and cell proliferative properties. Further investigation is warranted to isolate, confirm and characterize phytochemicals that are responsible for the medicinal properties observed.

## Introduction

The concept of combining dietary constituents to manage various illnesses is historically part of most of the cultures. Currently, there is wide spread use of complementary and alternative medicine (CAM) globally. Of five categories of CAM identified, the herbal products are the most abundantly used form of the therapy. The search for new herbal products/drugs for different human ailments is increasing, as they are believed to be less or non-toxic in nature. It is well recorded that various plants belonging to Cucurbitaceae family are used as herbal medicine in most of the culture. *Coccinia* plants (Ivy) belonging to Cucurbitaceae family have their own importance in traditional medicines, including Ayurveda, practiced in India, Chinese herbal medicines practiced in China and Unani system of medicine or Greco-Arab medicine practiced in Iran (Khan et al., 1979 ▶). 

Ivy gourd is known in India by various vernacular names like tondi in Marathi, Tindora, Tinda and Kundu in hindi, dondakaya in telugu, tomdekayi in kannada, etc. (Ali et al., 2005 ▶). In other parts of the world, it is known as Hong gua in Chinese, Bat in Vietnam, Pepasan in Malay, Yasai, Karasuuri in Japanese, Gourds ecarlate de l'Inde in France, pepino Cimarron in Spanish and Skariagenagurk. Ivy gourd (*Coccinia grandis*) is found in tropical Asia (India, Pakistan, Bangladesh, Sri Lanka, Indonesia, Malaysia, the Philippines, and Thailand), and Africa (Cooke, 1903 ▶). *Coccinia indica* Wright and Arn., and *Coccinia cordifolia *(L.) Cogn.,* Cephalandra indica*, Naud., and *Bryonia cordifolia* (L.) Voigt.( Kirtikar and Basu, 1994 ▶; Philcox,1997 ▶; Nagare et al., 2015 ▶) are the other names of * C. grandis* which is a climber, trailer, dioecious, and perennial plant. Young, tender and long slender stem tops, leaves, and tuberous roots of *C. grandis* are cooked or used as a seasoning and young fruits are used in salads. Every part of this plant has been considered of having some medicinal value (Yadav et al., 2010 ▶; Nagare et al., 2015 ▶). Plant preparations from *C. grandis* are indigenously used for various skin diseases, bronchitis, anorexia, cough, asthma, catarrh, and epilepsy. Moreover, in Unani systems of medicine it has been used for ringworm, psoriasis, small pox and scabies (Kirtikar and Basu, 1994 ▶; Philcox, 1997 ▶; Nagare et al., 2015 ▶). Plant preparations are also used for itchy skin eruptions, wound healing, leprosy, gonorrhea, pyelitis, cystitis, snakebite, malarial infection, infective hepatitis, and jaundice; Also, it has been given as a hepatoprotective remedy and for treating renal calculi (Kirtikar and Basu, 1994 ▶; Vadivu et al., 2008 ▶; Shaheen et al., 2009 ▶; Deshpande et al., 2011a ▶; Dnyaneshwar and Patil, 2011 ▶; Ramakrishnan et al., 2011 ▶; Sood et al., 2012 ▶). *C. grandis* is also known for its anti-diabetic, anti-obesity, antimicrobial, antifungal, antileishmanic, antioxidant, antihypertensive, antitussive, antiulcer, analgesic, antipyretic, antianaphylactic, and anti-cancer properties (Kirtikar and Basu,1994 ▶; Yadav et al., 2010 ▶; Tamilselvan et al., 2011 ▶; Pekamwar et al., 2013 ▶; Gill et al., 2014 ▶; Nagare et al., 2015 ▶). However, its therapeutic efficacy is yet not conclusive due to the lack of carefully controlled scientific investigations. Although, most of the studies have either used extract of stem, root and most often leaf alone or in combination, only few studies tested medicinal properties of *C. grandis* fruits (Vadivu et al., 2008 ▶; Shaheen et al., 2009 ▶; Deshpande et al., 2011a ▶; Dnyaneshwar and Patil, 2011 ▶; Ramakrishnan et al., 2011 ▶; Sood et al., 2012 ▶). To our knowledge, there are hardly any *in-vitro* studies testing cell proliferative property of *C. grandis* fruit. The goal of this study was to conduct preliminary phytochemical screening and to evaluate the potential antimicrobial, antioxidant and cell proliferative property of *C. grandis* fruit.

## Materials and Methods

**Plant extract**

Un-ripened fresh fruits of *C. grandis* were collected in June 2014 from a local Indian grocery store in Schaumburg, IL (USA) and authenticated at Department of Biology, Chicago State University in Chicago, IL. Fresh fruits were washed with distilled water to clean any debris. Fruits were cut into small pieces and dried at room temperature away from direct sunlight. Dried material was weighed and subjected to cold extraction using ethanol (94-96 %, BDH) and acetone at 4^o^C, for 48 hr with occasional stirring. Similarly, hot extraction was carried out using a Soxhlet apparatus (58^o^C; 7 cycles) with alcohol and acetone. Residues obtained on extractions were evaporated to dryness using ‘Rotavap’ and dried. All extracts were stored at 4^o^C until further use.

**Phytochemical screening:**

Dried fruit powder was extracted (at 4^o^C) for 48 hr in MilliQ water and obtained residue was dissolved in small volume of MilliQ water and labeled as “aqueous extract”. This aqueous extract along with ethanol and acetone extracts were subjected to preliminary phytochemical screening using following tests (Egwaikhide and Gimba, 2007 ▶; Roopashree et al., 2008 ▶; Abba et al., 2009 ▶; Njoku and Obi, 2009 ▶; Yadav and Agarwala, 2011 ▶; Sood et al., 2012 ▶; Soloman et al., 2013 ▶).

*Alkaloids*

Nine drops of 1% diluted hydrochloric acid was added to 6mL of extracts, mixed well and the mixture was left for some time and filtered. The filtrate was divided in three tubes (approx. 2 mL) and used for alkaloid testing as follows:

*a. Draggendorff’s Reagent*: Few drops of this reagent were added to filtrate; appearance of reddish brown/orange precipitates indicated the presence of alkaloids.

*b. Mayer’s reagent*: Filtrate was treated with few drops of *Mayer’s reagent* (potassium mercuric iodide). Formation of yellow creamy precipitates indicated the presence of alkaloids.

*c. Wagner’s reagent*: Filtrate was treated with 4-6 drops of *Wagner’s reagent* (iodine in potassium iodide). Formation of brown/reddish brown precipitation or coloration indicated the presence of alkaloids. 

*Carbohydrates (Molisch’s test)*

Few drops of *Molisch’s* reagent were added to 2 mL of extract. This was followed by gradual addition of 2 mL of concentrated sulfuric acid down the side of the test tube. The mixture was then allowed to stand for two to three minutes. Formation of red or dull violet color at the interface of the two layers indicated the presence of carbohydrates. 

*Flavonoids*

One mL of 10% lead acetate solution was added to 1 mL of the aqueous extract. The formation of a yellow precipitate indicated the presence of flavonoids.

*Cardiac glycosides (Keller-Killiani test)*

Crude extract (2-5 ml) was mixed with 2 ml of glacial acid containing 1-2 drops of 2% solution of ferric chloride. The mixture was then poured into another test tube containing 2 ml of concentrated sulfuric acid. A brown ring of a deoxy-sugar, characteristic of alcoholic cardenolides at the interface indicated the presence of cardiac glycosides. 

*Tannins (Braymer’s Test)*

To 5ml of the extract, few drops of 0.1% ferric chloride were added. A brownish green or a blue-black coloration indicated the presence of tannins. 

*Phlobatannins*

Two ml of hydrochloric acid 1% was added to 2 ml of the aqueous extract, the mixture was then boiled. Appearance of red precipitate indicated the presence of phlobatannins.

*Terpenoids (Salkowski test)*

Five ml of the extract was mixed with 2ml of chloroform and to that 3ml of concentrated hydrochloric acid was carefully added to form a layer. Presence of terpenoids is indicated by interface forming a reddish brown color. 

*Proteins/Amino acids*

Two ml of the extract was treated with 2-5 drops of 1% ninhydrin solution in acetone in a test tube and placed in boiling water bath for 1-2 min. Presence of amino acid was indicated by the formation of purple color. 

*Resins*

To 4mL of the extract, 4mL of 1% aqueous hydrochloric acid was added. Formation of resinous precipitate indicated the presence of resins.

*Saponins*

Three mL of extract was shaken vigorously with 2mL of distilled water and then, heated to boil; appearance of stable, persistent creamy miss of small bubbles indicated the presence of saponins. 

*Steroids (Liebermann Burchard Reaction)*

To 10 mL of extract, 10 ml of chloroform was added and filtered. In 2 mL of filtrate, 2 mL of acetic anhydride was added followed by few drops of concentrated sulfuric acid. The appearance of blue, bluish-green or a rapid change from pink to blue color/ring indicated the presence of steroids.

**Antibacterial activity assay**

Pure bacterial culture, nutrient broth, and culture plates were procured from *Carolina Biological Supply Company *(USA). The antibacterial activities of the cold and hot ethanol and acetone extracts were tested against two Gram positive (*Staphylococcus aureus *and* Enterococcus faecalis*) and two Gram-negative microorganisms (*Escherichia coli* and *Pseudomonas aeruginosa*) using modified *Kirby-Bauer* diffusion method. Bacterial strains, stored in *Muller-Hinton* broth, were sub-cultured for testing in the same medium and were grown at 37^º^C for 48 hr. Freshly cultured bacteria (100l, approx. 10^5^ bacteria) were used to inoculate the culture plates. Wells of 5mm diameter were made using sterilized glass pipette end. Various concentrations of the extracts in triplicates were added to the wells. Ethanol and acetone controls and antibacterial drug erythromycin (5mg/ml) used as a standard, were tested simultaneously with different extracts. All plates were incubated at 37^º^C for 48 hr and diameter of ‘zone of inhibitions’ (ZOI) was measured using a transparent metric ruler under magnifying glass. The lowest concentration of various extracts and standard that inhibited the visible growth after 48 hr was considered as the minimum inhibitory concentration (MIC). 

**Antioxidant activity assay**

The antioxidant property of the extracts compared to the standard (Trolox) was measured using ‘Cayman’s Antioxidant Assay’ (Catalog No. 709001, Cayman Chemical Company). Total antioxidant capacity of the sample was measured by extracts ability of inhibiting oxidation of 2, 2’-azino-di-[3-ethylbenzthiazoline sulfonate]. Sample’s antioxidant capacity is compared with a water-soluble tocopherol analogue (Tolox), and reported as molar Trolox equivalents. Only cold and hot ethanol extracts were used for this assay. Assay procedure restricted the testing of acetone extracts due to its interference with the assay reagents. The absorbance of the extracts was read at 405 nm using ELISA plate reader (apDia, A.D. Touch ELISA Reader). Different concentrations of Trolox as described in the assay procedure were used to generate the standard curve for comparison. The antioxidant property of the extracts was calculated using the following equation. 

Antioxidant (mM) = Sample average absorbance – (y-intercept)/slope x dilution)

All samples were run as triplicates using 96 well plates provided with the assay kit. 

**Cell proliferation assay**

The Cell Titer 96^®^ Aqueous One Solution Cell Proliferation Assay (MTS) (Catalog No. 3582, Promega Corporation) was used to study cell proliferation property with MDA-MB 321 breast cancer cells. This is a colorimetric method for determining the number of viable cells in proliferation. Cells were cultured in DMEM (Dulbecco's Modified Eagle Medium) with 4.5 g/L glucose without L-Glutamine containing 10% FBS (Fetal Bovine Serum) and 1% PES (phenazineethosulfate). Cells were cultured in flask as adherent cells. Actively growing cells (passage 2) were used and 5,000 cells (in 50 μl media) were seeded in each well of 96 well plates and grown for 48 hr using only medium. After 48 hr, medium was aspirated from each well and cells were subjected to various concentrations of the extracts and controls (culture medium, ethanol and acetone only) in triplicates. This well plate was kept at 37^o^C for another 48 hr in incubator having 5% CO_2_, humidified atmosphere. Then, 20 µl of MTS [3-(4, 5-dimethylthiazol-2-yl)-5-(3-carboxymethoxyphenyl)-2-(4-sulfophenyl)-2H-tetrazolium] reagent was added to each well and cells were incubated for another 4 hr and absorbance was read at 490 nm using ELISA plate reader. Cell proliferation property of the extracts was determined by the quantity of ‘Formazan product’ measured by the absorbance at 490 nm, which was directly proportional to the number of living cells in culture according to the assay protocol. The Hemocytometer was used for cell counting.

**Statistical analysis**

Results of this study are expressed as means ± standard deviations where appropriate and in units suggested by the assay procedures. Data were analyzed for correlation and differences using unpaired student's t-test and one way analysis of variance (ANOVA) followed by *post-hoc* analysis with GraphPad Prism Ver. 5 and a p<0.05 was considered statistically significant.

## Results

*C.* *grandis *fruits were extracted using solvents of different polarity. Twenty-five grams of dried powder of *C. grandis* fruits yielded 3.8 g of residue on cold and 4 g with Soxhlet extraction with ethanol. Similarly, 25 g of dried powder yielded 2.75 g of residue with cold and 3.2 g of residue on hot Soxhlet extraction with acetone. The findings of preliminary phytochemical screening are presented in Table 1. Both aqueous and ethanol extracts tested positive for resins, saponins and terpenoids with the exception of aqueous extract, which tested positive for carbohydrates, cardiac glycosides and proteins, whereas, ethanol extract tested positive for alkaloids, flavonoids, tannins and phlobatannins. Acetone extract tested positive for flavonoids.

**Table 1:** Preliminary phytochemical screening of C. grandis fruit extracts

**Tests **	**Aqueous Extract**	**Ethanol Extract**	**Acetone Extract**
**Carbohydrates**	(+)	(-)	(-)
**Proteins**	(+)	(-)	(-)
**Alkaloids**	(-)	(+)	(-)
**Cardiac Glycosides**	(+)	(-)	(-)
**Flavonoids**	(-)	(+)	(+)
**Tannins**	(-)	(+)	(-)
**Phlobatannins**	(-)	(+)	(-)
**Resins**	(+)	(+)	(-)
**Saponins**	(+)	(+)	(-)
**Terpenoids**	(+)	(+)	(-)
**Steroids **	(-)	(+)	(-)

(+) Indicates Presence, (-) Indicates Absence

**Table 2:** Zone of Inhibitions with different extracts of C. grandis fruit

	**Mean Zone of Inhibitions against ** ***S. aureus*** ** (mm ± SD)**			
	**Cold Ethanol** **(1)**	**Hot Ethanol** ** (2)**	**Cold Acetone** ** (3)**	**Hot Acetone** ** (4)**
**A **	12.5 ± 0.4	14.7 ± 0.2 *** +++	15.2 ±0.3 *** ++	16.5 ± 0.5 ***
**B**	10.7 ± 0.6	13.3 ± 0.6 ***	13.0 ± 0.0 **	14.3 ± 0.6 ***
**C**	6.5 ± 0.4	11.3 ± 0.2 ***	12.0 ± 0.0 ***	12.2 ± 1.0 ***
**D**	5.8 ± 0.2	10.2 ± 0.2 *** ###	7.2 ± 0.8 *** ++	9.0 ± 0.5 ***
**Mean Zone of Inhibitions against ** ***E. faecalis *** **(mm ± SD)**				
**A **	8.5 ± 0.0	9.2 ± 0.2	9.7 ± 0.6 *** ++	11.2 ± 0.3 ***
**B**	7.5 ± 0.7	7.3 ± 0.2 ++	8.3 ± 0.3	8.8 ± 0.3 *
**C**	6.0 ± 0.0	6.2 ± 0.2	6.8 ± 0.8	6.8 ± 0.3
**D**	NZOI	NZOI	NZOI	5.7 ± 0.3
**Mean Zone of Inhibitions against ** ***E. coli*** ** (mm ± SD)**				
**A **	9.1 ± 0.1	11.8 ± 0.2 *** ++	12.2 ± 0.8 *** ++	14.2 ± 0.8 ***
**B**	8.5 ± 0.0	9.8 ± 0.2 * +++	10.7 ± 0.6 *** ++	12.3 ± 0.6 ***
**C**	7.0 ± 0.0	8.0 ± 0.0 ** +++	7.8 ± 0.3 *** +++	9.8 ± 0.3 ***
**D**	NZOI	NZOI	NZOI	7.5 ± 0.5
**Mean Zone of Inhibitions against ** ***P*** **. ** ***aerugenosa*** ** (mm ± SD)**				
**A **	8.2 ± 0.2	8.7 ± 0.2	9.0 ± 0.0	9.0 ± 1.0
**B**	7.7 ± 0.2	8.2 ± 0.2	7.8 ± 0.3	8.5 ± 0.5
**C**	6.2 ± 0.5	6.8 ± 0.6	7.3 ± 0.8	7.5 ± 0.5
**D**	NZOI	NZOI	NZOI	NZOI
**E **	30.0±0.5	28.5±0.6	27.0±0.5	25.0±0.5

A: 29.60 g, B: 14.80 g, C: 7.40 g, D: 3.70 g, E: Erythromycin (300 g), NZOI = No Zone of Inhibition.

Zone of inhibition differed significantly between all extracts and control (0 g/mL) and differences were significant at p value of <0.05 on ANOVA.

* p<0.5,

** p<0.01 and

*** p<0.001 compared to cold ethanol and other extracts.

++ p<0.01,

+++ p<0.001 compared between hot acetone and other extracts.

### p<0.001 compared between cold acetone and hot ethanol on *post-hoc* analysis and no significant differences were observed between various concentrations of different extracts and standard erythromycin. Controls were pure ethanol and acetone that showed no zone of inhibitions.

**Table 3:** MIC for different extracts of C. grandis fruit as compared to erythromycin as the standard

** Minimum Inhibitory Concentration (MIC) (g/ml)**				
**Type of Extracts** *	***S. aureus***	***E. faecalis***	***E. coli***	***P. aerugenosa***
**Cold Ethanol**	56.7 ±0.71	86.67 ±1.0	80.0 ±1.62	108.3 ±1.9
**Hot Ethanol **	55.0 ±0.12	83.3 ±1.12	66.7 ±0.88	96.6 ±1.62
**Cold Acetone**	50.0 ±0.55	81.6 ±0.35	63.3 ±0.1	93.3 ±1.2
**Hot Acetone**	43.3 ±0.8	63.3 ±0.45	62.5 ±0.15	70.0 ±1.36
**Erythromycin (Standard)** **	36.6 ±0.5	38.3 ±0.72	43.0 ±1.0	63.3 ±1.554

* 60 l loaded in each well;

** Erythromycin=5g/ml, used as positive control

**Figure 1:**
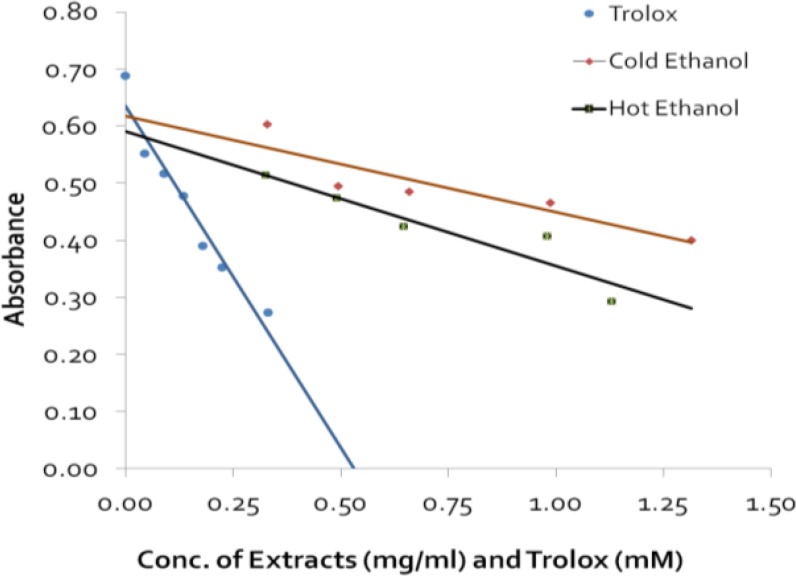
Comparison of the absorbance of cold and hot ethanol extracts of C. grandis fruit with Trolox

Results of antibacterial activity are presented in Table 2. Differences in zone of inhibitions among various concentrations of different extracts for *S*. *aureus*, *E*. *Faecalis and E. coli *were significant (p<0.05 to p<0.001) except for concentration of 7.40 g in case of *E. faecalis, *whereas, no significant differences in zone of inhibitions were observed against *P. aerugenosa *for various concentrations of all four extracts. Hot ethanol and acetone extracts exhibited greater zone of inhibitions for all four microorganisms tested compared to cold extracts. No zones of inhibitions were observed for concentration 3.70 g of cold and hot ethanol, and cold acetone extract against *E*. *faecalis, E. coli* and *P. aerugenosa *whereas, for hot acetone extract against *P. aerugenosa* only. The four extracts showed different levels of antibacterial activity. Zones of inhibitions for the standard (erythromycin) were highly significant to that of various concentrations of different extracts tested (p<0.05 to p<0.001) and one and a half to two times greater, which may have resulted because of the higher concentrations of the standard used. The mean MIC values were higher for *P. aerugenosa* ranging from 4.2 to 6.5 g and lower for *S. aureus* ranging from 2.60 to 3.4g for all four extracts tested. An increasing trend in mean MIC was observed with *S. aureus*, *E*. *Faecalis, E. coli*, and *P. aeurgenosa*. All extracts showed similar trend of antibacterial activity. The MIC for erythromycin also showed similar antibacterial trend (Table 3). Absorbance values of various concentration of Trolox, a known antioxidant, were used to plot a standard curve. Mean absorbance values of various concentrations of cold and hot ethanol extracts were compared to Trolox to determine the relative strength of antioxidant properties of the extracts (Figure 1). A decrease in absorbance with Trolox was considered as an evidence of increased antioxidant property indicating their inverse relationship. The antioxidant property measured by calculating Trolox equivalent of cold ethanol extract, was 0.197 mM for 1.315 mg/ml of the extract, 0.0263 mM for 0.329 mg/ml of the extract, whereas, the antioxidant capacity of hot ethanol extract was 0.287 mM for 1.13 mg/ml of the extract and 0.101 mM for 0.326 mg/ml of the extract (Table 4). The absorbance of cold and hot ethanol extracts were found strongly negatively correlated with that of standard Trolox (r= -0.96 and r= -0.99; p<0.05), respectively. Similarly, concentrations of cold and hot ethanol extracts were found strongly, negatively correlated with that of standard Trolox (r= -0.984 and r= -0.989; p<0.05) respectively.

**Table 4:** Antioxidant property of cold and hot ethanol extracts of C. grandis fruits expressed as Trolox Equivalent (TE

**Type of Extract**	**Conc. (**mg/ml**)**	**Mean** **Absorbance ±SD**	**Trolox Equivalent (TE)** *
**Cold Ethanol **	1.315	0.400 ± 0.024	0.197
	0.329	0.605 ± 0.062	0.0263
**Hot Ethanol **	1.13	0.293 ± 0.011	0.287
	0.326	0.515 ± 0.044	0.101

* Calculated using Antioxidant (mM) = sample average absorbance– (y-intercept)/slope x dilution).

The results of cell proliferation assay are presented in Table 5. Mean absorbance values for extracts were higher than any type of controls used. An increase in absorbance was considered as an evidence of absence of cytotoxic property indicating their inverse relationship. Significant differences were found in cell proliferation measured by absorbance among different concentrations of ethanol and acetone extracts compared to their control as well as among different extracts (p<0.05 to p<0.001). Cold extracts showed greater cell proliferation, suggesting increased cell growth compared to the hot extracts confirming the absence of any cytotoxic properties in *C. grandis* fruits. Cold acetone extract showed more marked results as a cell proliferative treatment than cold ethanol extract (Table 5 and Figure 2).

**Table 5:** Comparison of cell proliferation with different extracts of C. grandis fruit

**Absorbance (Mean ± SD)**						
**Conc.**	**Cold Ethanol** ** (1)**	**Hot Ethanol** ** (2)**	**Control for Ethanol (3)**	** Cold Acetone** ** (4)**	**Hot Acetone** ** (5)**	**Control for Acetone (6)**
**A **	1.302 ± 0.001 *** ++	1.271 ± 0.010	0.789 ± 0.010	1.415 ±0.030 $$$ ##	1.317 ± 0.018	0.770 ± 0.012
**B**	1.408 ± 0.028 *** +++	1.300 ± 0.016	0.788 ± 0.013	1.458 ± 0.032 $$$ ##	1.340 ± 0.020	0.772 ± 0.016
**C**	1.500 ± 0.030 *** +++	1.330 ± 0.019	0.778 ± 0.022	1.601 ± 0.022 $$$ ###	1.400 ± 0.010	0.766 ± 0.028

A. 4.94 g/10 µl+90 µL medium; B. 12.35g/25 µl+75 µL medium; C. 24.70 g/50 µl+50 µL medium Control

A: 10 µL+ 90 µL medium; B: 25µL+75µL medium; C: 50 µL+50 µL medium

Differences were significant at p value of <0.05 on ANOVA.

*** p<0.001 compared to control for ethanol with cold and hot ethanol.

++ p<0.01,

+++ p<0.001 compared to hot and cold ethanol.

$$$ p<0.001 compared to control for acetone with cold and hot acetone.

## p<0.01,

### p<0.001 compared to hot and cold acetone.

**Figure 2:**
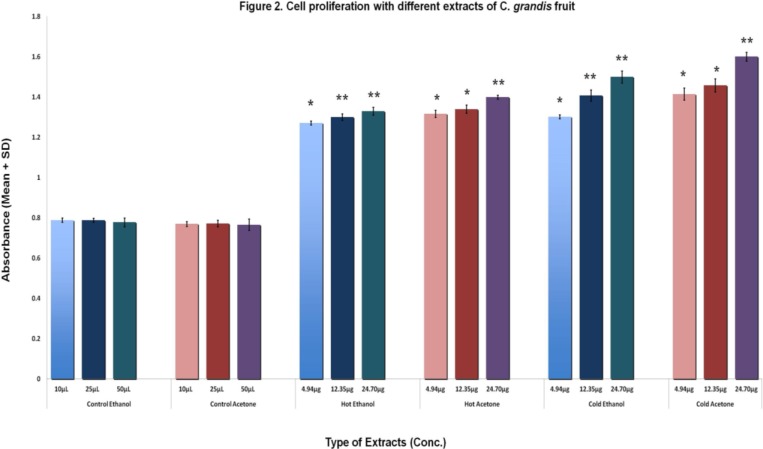
Cell proliferation with different extracts of C. grandis fruit. Data are presented as Mean ± SD; *Significant difference between extracts and controls on post-hoc analysis at p value of <0.05 and ** at p value of <0.001

## Discussion

It is well known that the various phytochemicals that are present in herbal medicines and plants are responsible for their medicinal values. Nature and extent of phytochemicals present in these plants explain their medicinal values. Phytochemicals are present in all parts of plants; however, it has been reported that the leaves, roots, bark and stems have higher concentration of such phytochemicals compared to fruits and flowers (Siddiqui et al., 2009 ▶). Phytochemicals that we found in the fruits are also found in other parts like leaves, stem, and roots of *C. grandis* (Tamilselvan et al., 2012 ▶; Gautam et al., 2014 ▶; Hossain et al., 2014 ▶). The antibacterial, antioxidant and cell proliferative properties of *C. grandis* fruits observed in this study, could be attributable to the presence of phytochemicals that were identified during preliminary screening. Phytochemicals such as alkaloids, terpenoids, glycosides, flavonoids, and tannins are known to possess antibacterial and antioxidant properties. Flavonoids were also reported to have a broad spectrum of medicinal properties such as antioxidant, anti-inflammatory, antimicrobial, anti-cancer and cardio-protective activities. Flavonoids are hydroxylated polyphenols found in plants known for their wide range of antimicrobial activity *in vitro*. Similar to tannins, flavonoids show their antibacterial activity by forming complex with extracellular and soluble proteins. In this study, both ethanol and acetone extracts tested positive for flavonoids. In addition, ethanol extract tested positive for alkaloids, tannins, resins, saponins, terpenoids and steroids. This may be the reason why ethanol extract exhibited greater antibacterial activity compared to other extracts. Tannins are naturally occurring plant polyphenols, which can be classified into hydrolysable and non-hydrolysable (condensed) tannin. The ability of plant polyphenols to form complex with minerals and polymers, has been suggested as the major mechanism of their antibacterial activity. This complex induced by tannins, is considered being toxic and inhibitory to microbial enzymes. Studies have suggested that this inhibitory mechanism involves direct interaction with membranes, extracellular proteins and cell walls. In this study, tannins may have also played a major role in inhibiting the growth of microorganisms tested. Shaheen et al. (2009) ▶ also reported similar phytochemicals testing positive in their study.

**Figure 3:**
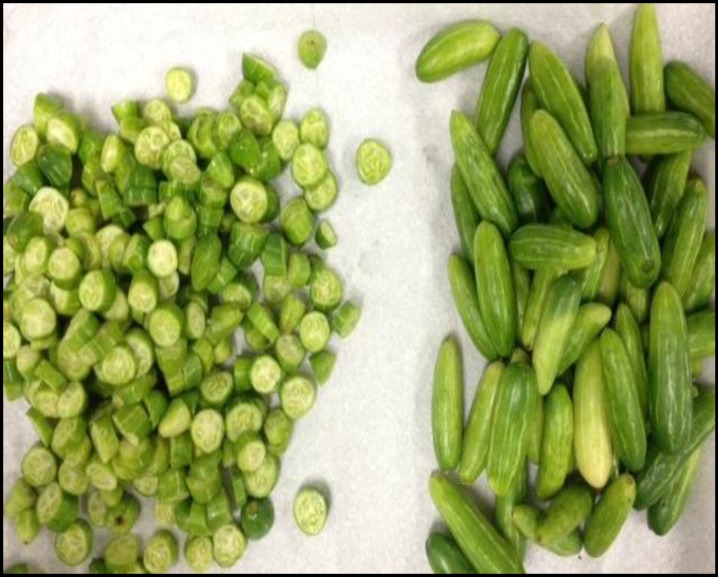
Picture of C. grandis fruits

Antibacterial properties of *C*. *grandis* leaves have been extensively studied (Farrukh et al., 2008 ▶; Bhattacharya et al., 2010 ▶; Hussain et al., 2010 ▶; Bulbul et al., 2011 ▶; Satheesh and Murugan, 2011 ▶; Sivraj et al., 2011 ▶; Khatun et al., 2012 ▶). Moreover, study by Shaheen et al. (2009) ▶ has reported similar activity for fruits of *C*. *grandis. * In this study, hot petroleum ether, diethyl ether, chloroform, ethyl acetate, acetone, methanol and ethanol extracts were used for screening the antibacterial activity whereas, in our study, we used both cold and hot extracts of ethanol and acetone. Antibacterial activities observed in our study, were comparable to those of ethanol and acetone extracts studied by Shaheen et al. (2009) ▶. However, in their study, petroleum ether and methanol extracts showed more pronounced antibacterial activity against gram-positive organisms including *S. aureus*, being more susceptible and *S. paratyphi* A, being more resistant, whereas least activity was observed with chloroform extract (Shaheen et al., 2009 ▶). In contrast, in our study, acetone extract exhibited higher antibacterial activity than the ethanol extract and hot extracts were more effective than the cold ones. The choice of different solvents and bacteria used for antibacterial activity testing also limited our ability to make appropriate comparisons for antibacterial activities. In a study by Sivaraj et al. (2010) ▶, ethanol extract of leaves showed higher antibacterial activity against *E. coli* compared to acetone or methanol; however, we found acetone extract to be more potent in inhibiting growth of all gram-positive and gram-negative bacteria (including *E*. *coli*), than the ethanol extract (Sivraj et al., 2010 ▶). The differences in MIC were profound for *S. aureus* and *E. coli* for fruit extracts, being lower compared to MIC values of alcohol and acetone extracts of leaves. The MIC reported by Sivaraj et al. (2010) ▶ for acetone extract against *S*. *aureus* and *E*. *coli *were 1000g/mL and 500 g/mL, respectively, in contrast to the ones that we observed for cold and hot acetone extract against *S*. *aureus* (50 g/mL and 43.3 g/mL), and *E*. *coli *(63.3 g/mL and 62.5 g/mL), respectively. These differences in MIC could be attributed to the differences in plant material and the type and amount of phytochemicals present in them. Higher MIC for ethanol extracts of *C*. *grandis* leaves against *S. aureus* (1750 µg/mL), *E*. *coli* (1500 µg/ml) and *P*. *aeruginosa* (1500 µg/ml) reported by Bhattacharya et al. (2010) ▶. It would be difficult to compare MIC values reported in other studies, as they differ based on the solvent used as well as the bacterium. Based on the above observations, we can safely presume that the fruits possess greater antibacterial activity than the leaves. 

Free radicals are known to play a vital role as etiological factors in a wide variety of pathological conditions including diabetes, autoimmune disorders, aging, cardiovascular diseases, neurodegenerative diseases, etc. Antioxidants are agents, which scavenge free radicals; thereby, they prevent damages to proteins, enzymes, carbohydrates, DNA and lipids (Halliwell and Gutterridge, 1999 ▶; Fang et al., 2002 ▶; Lee et al., 2004 ▶). Lee et al. (2004) ▶ classified antioxidants into two major categories namely, enzymatic and non-enzymatic. The enzymatic antioxidants are produced endogenously and include superoxide dismutase, glutathione peroxidase and catalase. The non-enzymatic category includes some phytochemicals such as curcumin, ascorbic acid, carotenoids, tocopherols, tannins, flavonoids that are mostly obtained from plant sources. The results of our study indicate that the fruits of *C. grandis* are a potential source of natural antioxidants. The antioxidant assay used in this study measured the total antioxidant capacity of the sample. Our extracts detected the presence of more than one phytochemical suggesting that this activity can be attributed to the presence of both tannins and flavonoids. Our ethanol extract also exhibited antioxidant activity, showing strong linear relationship with the concentrations of extract tested, compared to Trolox. Several studies have reported antioxidant property of *C*. *grandis* leaves, stem and root extracts (Nanasombat and Teckchuen, 2009 ▶; Bulbul et al., 2011 ▶; Deshpande et al., 2011a ▶; Moideen et al., 2011 ▶; Ashwini et al., 2012 ▶; Bhaduria et al., 2012 ▶; Maheshwari et al., 2015 ▶); however, none of these studies tested this activity in fruits. Antioxidants are known for being able to reduce inflammation. *C*. *grandis* extracts are used as herbal medicine for various illnesses including inflammation. This further proves why *C*. *grandis* is used and known for its anti-inflammatory properties in folklore and Indian traditional medicine, Ayurveda (Junaid et al., 2009 ▶; Niazi et al., 2009 ▶; Deshpande et al., 2011b ▶; Ashwini et al., 2012 ▶). 

Bulbul et al. (2011) ▶ has reported adverse effects of various *Coccinia* extracts on cell survival. Studies have also reported partial cure of gastric ulcer with leaf extract (Papiya et al., 2008 ▶; Preeth et al., 2010 ▶) as well as its anticancer/anti-tumor properties (Bhattacharya et al., 2011 ▶; Girish et al., 2011 ▶; Behera and Dash, 2012 ▶). Bunkrongcheap et al. (2014) ▶ conducted an *in vitro* cell culture study in 3T3-L1-cell using root extract of *C*. *grandis*. This study examined the effects on differentiation of pre-adipocytes, in contrast to ours, where we examined the cell proliferative property (cytotoxicity) using MDA-MB-231 breast cancer cells. In contrary to findings from other studies, we found that the number of cells increased following incubation with all four extracts. Our results indicated that cold extracts were better than the hot extracts and acetone extract was better than ethanol in promoting cell proliferation; however, it indicated the absence of any cytotoxic properties in *C. grandis* fruits. This cell proliferation property could be attributed to steroidal hormone-like compound(s) present in these fruits, which needs to be further investigated. It will be interesting to study the cell proliferation process in normal (non-cancerous) cells using primary cell culture method in future investigations. 

Our study had some limitations. We did not use isolated and characterized phytochemicals from *C*. *grandis* fruits for confirming its antibacterial, antioxidant and cell proliferative properties. Since, the extracts may have contained several phytochemicals and were used for screening of these potential activities, there is a possibility that they may have interfered with the assay procedures used and the readings obtained. 

The findings of this study suggest that *C*. *grandis* fruit contain alkaloids, flavonoids, tannins and steroids, which perhaps responsible to some degree for its antibacterial, antioxidant and cell proliferative properties as mentioned in folklore and Indian traditional medicine, Ayurveda. However, its cell proliferative property deserves further investigation. Comparison of cell proliferation behavior among cancerous versus non-cancerous cells could be an interesting topic for future investigations.
